# Editorial for the Special Issue: Pharmacokinetics of Orally Administered Drugs, 2nd Edition

**DOI:** 10.3390/pharmaceutics16081078

**Published:** 2024-08-17

**Authors:** Márcio Rodrigues, Gilberto Alves

**Affiliations:** 1 CPIRN-UDI-IPG—Center for Potential and Innovation of Natural Resources, Research Unit for Inland Development, Polytechnic Institute of Guarda, Av. Dr. Francisco de Sá Carneiro, 6300-559 Guarda, Portugal; marciorodrigues@ipg.pt; 2 CICS-UBI—Health Sciences Research Centre, University of Beira Interior, Av. Infante D. Henrique, 6200-506 Covilhã, Portugal

## 1. Introduction

Oral administration of medicines has been the most common route for drug delivery as it is cost-effective and is convenient for patients [1,2]. In fact, during the drug discovery process, a frequent goal is the search for drug candidates that enable an adequate systemic exposure via the oral route [2]. However, there are several factors that limit the oral bioavailability, such as poor aqueous solubility, hydrolysis of the drug in the gastrointestinal tract, low permeability, dissolution rate, particle size, extensive intestinal and hepatic first-pass metabolism, high affinity for efflux transporters, and the food effect, among others [3,4]. Thus, the focus of this Special Issue was to gather scientific information regarding the latest evidence and recent advances in this field. This was achieved as the current Special Issue contains six original articles devoted to the development of (nano)pharmaceutical approaches to improve the bioavailability and pharmacokinetic profile of orally administered drugs.

## 2. Overview of the Published Articles

In this context, it is common to first conduct non-clinical studies in rodent models (mice and/or rats) to obtain a proof-of-concept of the feasibility of an idea or a proposed new product. In one of these studies tenofovir disoproxil (TD), an ester prodrug of tenofovir that has been marketed as tenofovir disoproxil fumarate (TDF) to avoid the hydrolysis of TD due to moisture, was developed in a different form (i.e., the stability-enhanced solid-state TD free base crystal—SESS-TD crystal) aiming at improving solubility in gastrointestinal tract and stability. Based on the comparable pharmacokinetic profiles of tenofovir obtained in rats after the oral administration of the SESS-TD crystal form and the marketed TDF form, the feasibility of SESS-TD crystal was demonstrated to be used as an active pharmaceutical ingredient for tenofovir instead of TDF (contribution 1). In another study, it was shown that the febuxostat/L-pyroglutamic acid cocrystal resulted in an increased bioavailability in rats and mice, when compared with febuxostat (contribution 2). At this point, it should also be highlighted that Kim et al. (contribution 3) showed that co-amorphous dispersions of mirabegron, namely mirabegron-1,2-ethanedisulfonic acid, mirabegron-1,5-naphthalenedisulfonic acid, and mirabegron-*L*-pyroglutamic acid, resulted in an increase in the bioavailability in rats and mice compared with the mirabegron. On the other hand, Wang et al. (contribution 4) tested a liquid formulation containing nirmatrelvir and ritonavir, by adopting strategies that used co-solvents and surfactants to enhance the solubility and inhibit possible recrystallization to be an alternative to Paxlovid^®^ tablets for patients that have difficulties with swallowing (e.g., the elderly and those with dysphagia). The results obtained in rats showed that the oral bioavailability of nirmatrelvir and ritonavir solution was significantly enhanced compared to Paxlovid^®^ tablets; in particular, the AUC_0–t_ of nirmatrelvir and ritonavir increased by 6.1 and 3.8 times, respectively, while the C_max_ increased by 5.5 times for both drugs. In addition, the liquid formulation was shown to be physically and chemically stable at 4 °C, 25 °C, and 40 °C for 90 days.

In another study, a bioanalytical method was developed using liquid chromatography coupled to tandem mass spectrometry to assess the pharmacokinetics and tumor distribution of fenretinide, a synthetic retinoid chemically related to all-trans-retinoic acid, after administration of a novel oral nanoformulation of fenretinide. This novel method was used to support preliminary pharmacokinetic studies, after acute and chronic oral administration of the nanoformulation, with fenretinide being detected in plasma and tumor tissue at concentrations higher than the IC_50_ value necessary for in vitro inhibitory activity (i.e., 1–5 µM) in different cancer cell lines. In addition, active and inactive metabolites of fenretinide were also detected in plasma and tumor homogenate (contribution 5).

Finally, a clinical study was also published in this Special Issue, which was conducted in healthy adult subjects, using a cross-over design, aiming at evaluating the performance of a new formulation of acetylsalicylic acid mini-tablets in comparison with a reference powder formulation. The results obtained were comparable, suggesting that the use of acetylsalicylic acid mini-tablets is feasible and, when required, may also improve medication adherence in children (contribution 6).

## 3. Conclusions

The papers presented in this Special Issue are of interest to the global scientific community in the field of oral drug administration and could be a valuable guide to the application of new oral formulations in clinical practice. 

We would like to thank the reviewers and editorial team who made significant contributions to this Special Issue in *Pharmaceutics*.
